# Preventing diabetes in obese Latino youth with prediabetes: a study protocol for a randomized controlled trial

**DOI:** 10.1186/s12889-017-4174-2

**Published:** 2017-03-16

**Authors:** Erica G. Soltero, Yolanda P. Konopken, Micah L. Olson, Colleen S. Keller, Felipe G. Castro, Allison N. Williams, Donald L. Patrick, Stephanie Ayers, Houchun H. Hu, Matthew Sandoval, Janiel Pimentel, William C. Knowler, Kevin D. Frick, Gabriel Q. Shaibi

**Affiliations:** 1 Center for Health Promotion and Disease Prevention, College of Nursing and Health Innovation, Arizona State University, 500 N. 3rd Street, Phoenix, AZ 85013 USA; 2 Family Wellness Program, Virginia G. Piper, St. Vincent de Paul Medical and Dental Clinic, 1730 E. Monroe Street, Phoenix, AZ 85034 USA; 3 Division of Endocrinology and Diabetes, Phoenix Children’s Hospital, 1919 East Thomas Road, Phoenix, AZ 85016 USA; 4 Southwest Interdisciplinary Research Center, Arizona State University, 411 N. Central Avenue, Suite 720, Phoenix, AZ 85004-0693 USA; 5 Seattle Quality of Life Group, Department of Health Services, School of Public Health and Community Medicine, University of Washington, Seattle, USA; 6 Department of Radiology, Phoenix Children’s Hospital, 1919 East Thomas Road, Phoenix, AZ 85016 USA; 7 Valley of the Sun YMCA, 350 N. 1st Avenue, Phoenix, AZ 85003 USA; 8 Diabetes Epidemiology and Clinical Research Section, National Institute of Diabetes and Digestive and Kidney Diseases, National Institutes of Health, 4212 North 16th Street, Phoenix, AZ 85016 USA; 9 Johns Hopkins Carey Business School, 100 International Drive, Baltimore, MD 21202 USA

**Keywords:** Adolescents, Latino, Obesity, Diabetes prevention, Intervention, Disparities

## Abstract

**Background:**

Obese Latino adolescents are disproportionately impacted by insulin resistance and type 2 diabetes. Prediabetes is an intermediate stage in the pathogenesis of type 2 diabetes and represents a critical opportunity for intervention. However, to date, no diabetes prevention studies have been conducted in obese Latino youth with prediabetes, a highly vulnerable and underserved group. Therefore, we propose a randomized-controlled trial to test the short-term (6-month) and long-term (12-month) efficacy of a culturally-grounded, lifestyle intervention, as compared to usual care, for improving glucose tolerance and reducing diabetes risk in 120 obese Latino adolescents with prediabetes.

**Methods:**

Participants will be randomized to a lifestyle intervention or usual care group. Participants in the intervention group will attend weekly nutrition and wellness sessions and physical activity sessions twice a week for six months, followed by three months of booster sessions. The overall approach of the intervention is framed within a multilevel Ecodevelopmental model that leverages community, family, peer, and individual factors during the critical transition period of adolescence. The intervention is also guided by Social Cognitive Theory and employs key behavioral modification strategies to enhance self-efficacy and foster social support for making and sustaining healthy behavior changes. We will test intervention effects on quality of life, explore the potential mediating effects of changes in body composition, total, regional, and organ fat on improving glucose tolerance and increasing insulin sensitivity, and estimate the initial incremental cost effectiveness of the intervention as compared with usual care for improving glucose tolerance.

**Discussion:**

The proposed trial builds upon extant collaborations of a transdisciplinary team of investigators working in concert with local community agencies to address critical gaps in how diabetes prevention interventions for obese Latino youth are developed, implemented and evaluated. This innovative approach is an essential step in the development of scalable, cost-effective, solution oriented programs to prevent type 2 diabetes in this and other populations of high-risk youth.

**Trial Registration:**

NCT02615353, registered on June 8, 2016.

## Background

Obesity is a significant public health problem and adolescence represents a critical life stage for obesity-related disease and prevention [1]. Latino adolescents are disproportionately impacted by several obesity-related chronic diseases including type 2 diabetes (T2D) [2, 3]. Latino youth exhibit higher rates of insulin resistance and prediabetes compared with white youth. These disparities support projections that 50% of Latino youth will develop T2D in their lifetime [2, 4–9]. Furthermore, conversion from prediabetes to T2D is accelerated during adolescence due to pubertal insulin resistance. This highlights the need for diabetes prevention programs during this critical transition period [10–12]. In addition to physical health consequences, obesity among Latino youth is associated with significant psychosocial maladjustments leading to lower quality of life (QoL) and increased risk for premature mortality in adulthood [13–15]. Given that Latino youth are the fastest growing segment of the pediatric population in the U.S., there exists a significant need for intensive and culturally-tailored T2D prevention efforts for this vulnerable group [16].

Compared to adults, the evidence base for T2D prevention and treatment in youth is limited [17]. The Diabetes Prevention Program (DPP) established that T2D can be prevented or delayed through intensive lifestyle interventions in adults with prediabetes [18–20]. To date, there are no T2D prevention studies for prediabetic Latino youth [21–23]. A recent review of intervention studies on obesity-related health disparities in minority youth found that the most successful programs were intensive lifestyle interventions that incorporated family, were culturally tailored, and utilized a multi-level model approach [24, 25]. However, many of these studies did not include high-risk populations, relevant T2D outcomes, nor sufficient follow-up periods, thus limiting the ability to draw conclusions regarding diabetes prevention [25, 26].

**Fig. 1:**
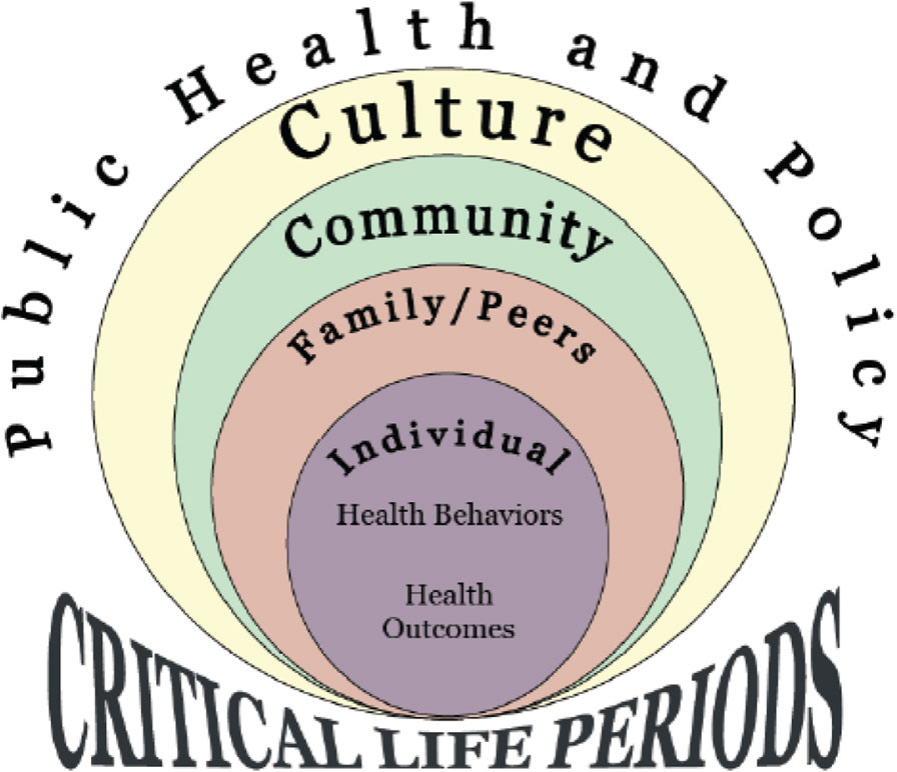
Expanded Ecodevelopmental Model

From a systemic perspective, programs for obese youth have historically focused on influencing individual-level factors, thus limiting their effectiveness in terms of sustainable behavior change and weight loss [27, 28]. Individual behaviors among obese youth occur within the context of a multi-level environment that includes, among other things, relationships with family and friends; opportunities and challenges in the community; cultural beliefs, practices, and customs; as well as access to healthcare services. These contextual influences are important for supporting and maintaining individual behavior changes in youth and are critical during adolescence when information processing occurs through multiple channels of influence [29–31]. The Expanded Ecodevelopmental Model (EEM; Fig. 1) is a framework that maps the complex, multi-level relationships that occur between individual, peer, family, and community-level factors. This model takes into consideration that youth exist within families and that interfamilial and contextual factors such as socioeconomic status, family connectedness, or conflict, influence health behaviors in youth and the family network. Furthermore, community-level factors such as physical activity and diet resource availability, crime, and neighborhood safety, influence family decision making and ultimately youth health behaviors [32]. The EEM model holds that these sources of influence can be leveraged to promote and support changes in health behaviors and health outcomes during critical life transition periods such as adolescence [33, 34]. Furthermore, successful diabetes prevention programs in adults draw heavily from Social Cognitive Theory (SCT) to support nutrition and physical activity behavior change [35, 36]. Applying elements of SCT is recommended for community-based interventions, although few studies with obese youth have been based on well-established socio-behavioral theory, nor have they been implemented in a community setting to support diabetes prevention [37, 38]. The current research study and its team have adapted the EEM as a guiding framework to develop diabetes prevention strategies, as informed by SCT, and as applied to Latino youth. We have applied this systemic model to develop, pilot test, and expand a culturally-grounded, community-based diabetes prevention program for obese Latino adolescents [39].

We have now further refined the model to meet the specific needs of obese Latino youth with prediabetes. This manuscript describes the National Institutes of Health funded randomized controlled trial (RCT) that was designed to examine the effectiveness of a culturally-grounded diabetes prevention program for obese Latino youth with prediabetes, as compared to a usual care control (UCC) group. This study will test the short-term (6-month) and long-term (1-year) efficacy of a lifestyle intervention that integrates nutrition, physical activity, and emotional health and well-being into a single curriculum that is delivered in the community. Proposed outcomes include changes in glucose tolerance (Specific Aim 1) and general and weight-specific QoL (Specific Aim 2), while exploring the mediating effects of changes in total, regional, and organ fat on T2D risk reduction (Specific Aim 3), and examining the initial incremental cost-effectiveness of the intervention to improve glucose tolerance (Specific Aim 4).

## Methods

### Study design

**Fig. 2:**
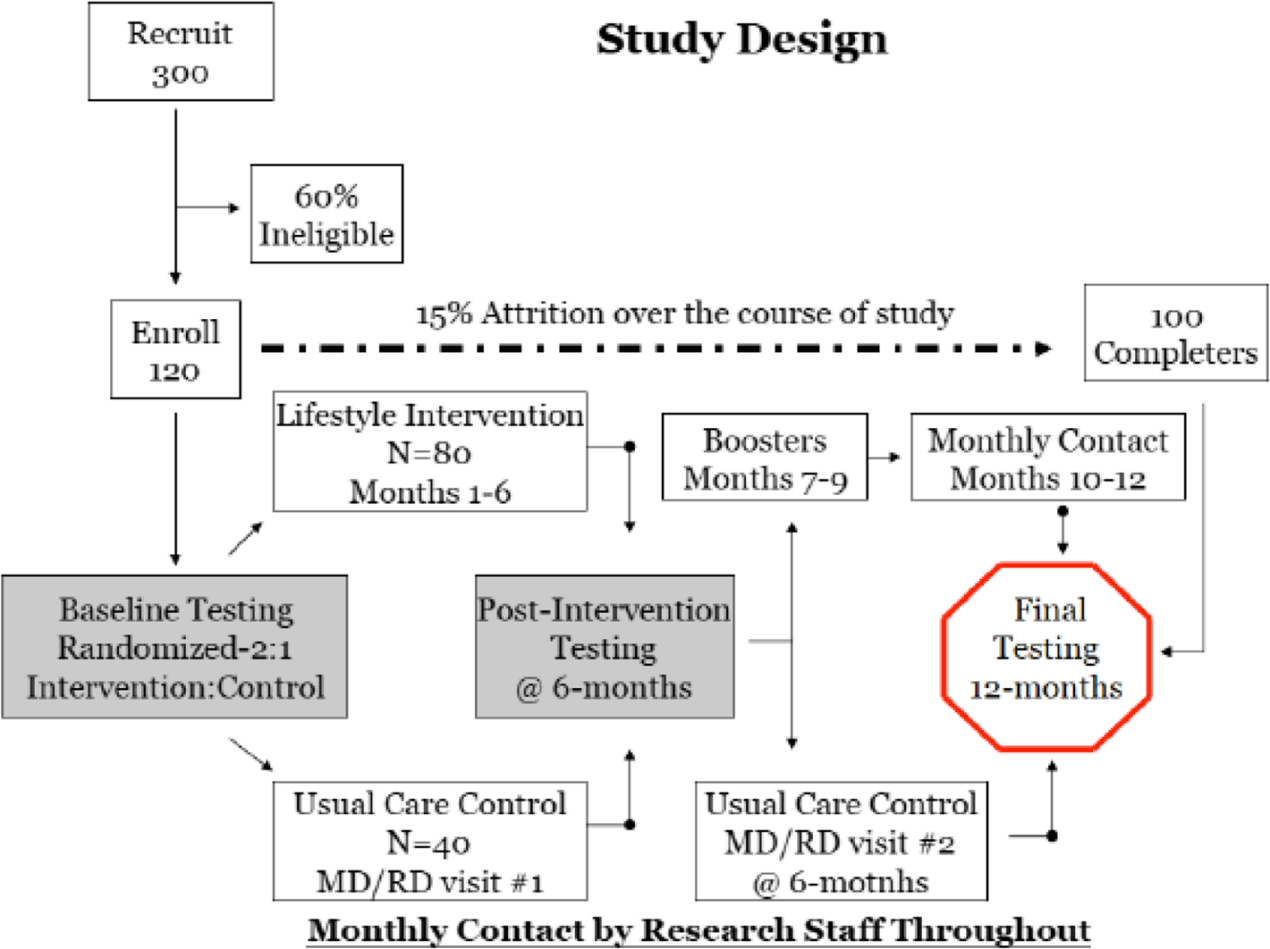
Study Design

This RCT will include 120 obese Latino adolescents aged 12–16 with prediabetes. Participants will be randomized (2:1 randomization) to the intervention or control condition. Participants in the intervention condition (*N* = 80) will attend weekly nutrition and wellness group education sessions (about 1.5 h each) led by trained health educators and two moderate to vigorous, group physical activity (PA) sessions per week (about 1-hour each) led by trained physical activity instructors at a YMCA located in central Phoenix, AZ. In addition, youth will be instructed to perform an additional hour of moderate to vigorous physical activity with a family member or friend outside of class. Participants randomized to the UCC condition (*N* = 40) will meet with a pediatric endocrinologist and a registered dietitian at baseline and at 6 months. The health educators will also provide standard lifestyle counseling regarding healthy eating and physical activity. Youth randomized to UCC will receive a YMCA membership and an abridged version of the program after their 12-month visit. All participants will undergo data collection at baseline, 6-months, and 12-months (Fig. 2).

### Participants

We will recruit equal numbers of males and females who meet the following specific inclusionary and exclusionary criteria: 1) self-identify as Latino, 2) between the ages of 12–16 years at enrollment, 3) obese, defined as a BMI ≥95^th^ percentile for age and sex or a BMI ≥30 kg/m^2^, and 4) prediabetic, defined as an HbA1c ≥5.7%, or fasting glucose ≥100 mg/dl, or 2-hour glucose ≥120 mg/dl. This inclusion criterion for prediabetes is in line with that used by the American Diabetes Association, with the exception of the post prandial glucose levels. The rationale for a lower post prandial glucose cut point stems from data in children that have shown youth with a 2-hour glucose of ≥120 mg/dl have similar diabetes risk, as defined by beta cell functioning, and similar rates of conversion to T2D as youth with a 2-hour glucose of ≥140 mg/dl, the formal definition used by America Diabetes Association [40, 41]. Participants will be excluded if they: 1) are taking medication(s) or diagnosed with a condition that influences carbohydrate metabolism, PA, and/or cognition, 2) have T2D, 3) have elevated liver enzymes (alanine aminotransferase ≥300 IU/L, aspartate aminotransferase ≥300 IU/L) or elevated triglycerides (>500 mg/dl), 4) have been hospitalized within the past two months, 5) are currently enrolled in a formal weight loss program, 6) have been diagnosed with depression or any other condition that may impact QoL, or 7) are pregnant. We will oversample and recruit 300 youth with the conservative assumption that 60% of those approached will be ineligible or not interested and 15% will be lost to attrition over the course of the study, thus leaving a proposed final sample size of 100 participants available for analysis at 12-months.

### Recruitment strategies

Recruitment will be coordinated through the Family Wellness Program at St. Vincent de Paul Medical and Dental Clinic (SVdP) in Phoenix, Arizona. The clinic provides primary and specialty services to Latino children and families and is an established and trusted entity in the local Latino community. SvdP’s referral network extends to more than 100 schools, community centers, and healthcare organizations in the greater Phoenix area. Recruitment information will be disseminated by distributing flyers to schools and community partners, hosting presentations, attending health fairs, and through direct contact/referral by providers. Additional recruitment activities driven by the research team will include formalizing mechanisms for referring patients from a large Latino serving Federally Qualified Health Center, smaller pediatric practices, and from Phoenix Children’s Hospital, the largest free-standing children’s hospital in the region.

### Ethics

The study protocol and all study-related documents will be approved and monitored by the Institutional Review Board at Arizona State University. All study-related documents will be available in English and Spanish with bilingual/bicultural research staff administering consent, collecting data, and answering questions. The study is registered at www.clinicaltrials.gov (Clinicaltrials.gov Identifier: NCT02615353).

### Procedures

#### Informed consent

Trained research team members will obtain written parental consent and child assent prior to any data collection procedures. Participants will be informed that their participation is voluntary and they are free to withdraw from the study at any time. Participants will also be informed that nonparticipation will not affect any health or medical services they currently receive and that confidentiality will be maintained.

#### Health screening & phenotyping

Potential participants will be invited to the ASU Clinical Research Unit for a health screening visit to determine eligibility. Potential participants will arrive at the ASU Clinical Research Unit at ~8:00 AM after an overnight fast for initial screening including a brief health history, height and weight measurements to calculate BMI percentile, and a standard 75 g oral glucose tolerance test (OGTT) to measure fasting and 2-hour glucose. Participants identified as obese and prediabetic during screening will be enrolled in the study and return within 3 months for baseline testing (described in detail below).

### Intervention overview

#### Group health education classes

The health education curriculum used in this intervention is informed by SCT and applies key behavioral change strategies from the adult DPP and other successful lifestyle interventions such as goal-setting, fostering social support, and enhancing self-efficacy to facilitate health behavior changes. In contrast to the DPP and other weight-management programs for obese populations, weight loss is not a primary goal of the intervention. The primary goals of the diabetes prevention intervention are: 1) making and sustaining healthy behavior changes (diet and physical activity), 2) enhancing QoL, and 3) reducing diabetes risks. The curriculum has been developed through an inductive, collaborative process with our community partners. The initial curriculum has been sequentially refined through a series of increasingly rigorous projects starting with a clinical demonstration project, a pilot study, and a recently completed randomized control trial [42–44].

This curriculum has been tailored to the unique psychosocial and developmental characteristics of obese adolescents and has been grounded in the Latino cultures to incorporate cultural relevance in addressing various Latino youth needs. These include promoting psychosocial well-being during this early-life developmental stage by fostering social support from friends and family, and include enhancing self-efficacy for making healthy behavior changes. Social support is fostered through appraisal from health educators as youth review OGTT results and set goals for reducing T2D risk, families exchange contact information and children exchange school information to facilitate interaction outside of the program. Furthermore, emotional well-being is incorporated throughout the curriculum with an emphasis placed on building self-esteem, positive self-affirmation, as these can aid in countering negative social influences imposed by family and peers. Self-efficacy is enhanced through setting and monitoring of health behavior goals, role playing and modeling of health behaviors, and through verbal encouragement from health educators, families, and peers. This curriculum is also grounded in Latino culture whereby curriculum content and its delivery is guided by the unique traditions, beliefs, and customs of the local Latino community. Curriculum delivery is also grounded by core Latino values that include: respect (respeto), trust (confianza), and social awareness in fostering close interpersonal relationships (simpatia) [45]. This curriculum is delivered in group classes using a family-based model. Latino families often exhibit shared values that emphasize the importance of the entire family (familismo) over any one individual, thus integrating the family into the intervention can. Also, this familial involvement aids in fostering social support for healthy behavior change [46]. Finally, this intervention is delivered by bilingual/bicultural community health educators. This grounds the intervention in a culturally-specific manner whereby health educators from the local community serve as a bridge in working with a minority and low-health literacy community. This approach has been recognized by the Centers for Disease Control and Prevention and the Institute of Medicine [47, 48].

Nutrition and wellness intervention sessions (*N* = 20) are led by health educators and delivered at the YMCA to groups of 8–10 families. A parent/guardian is required to participate in these sessions to further enhance social support from family members. Classes are delivered by bilingual/bicultural community health educators using a tiered approach where the first 16-sessions are delivered weekly while the last four sessions are distributed over a period of eight weeks. This delivery approach is designed to build self-sufficiency among these families as they become less reliant on the health educators and more independent in achieving their health and behavior goals. Participants are incentivized through a point system for attendance, participation in discussions, completing out of class ‘assignments’ such as helping to prepare a healthy meal for the family, and making progress towards their individual health goals. Parents and children work with other families during classes and are encouraged to do so outside of class in order to build a support network that extends beyond the program. A detailed intervention manual is used for intervention delivery and 25% of classes will be directly observed by a study team member using the manual and a checklist to ensure fidelity of delivery. Participant workbooks used for in and out of class activities will be reviewed to ascertain receipt of nutrition and well-being concepts.

##### Physical activity

The PA curriculum is led by YMCA fitness instructors and includes structured and unstructured components. Groups of 8–10 youth (both boys and girls) will engage in two classes per week for 60-minutes each. Classes include aerobic and resistance exercises delivered in a progressive manner with the first 2–4 weeks focusing on motor skill acquisition, exercise confidence, developing a fitness base, and building camaraderie among participants. Aerobic exercises include group activity classes (e.g. spinning and cardio kick-boxing) with the goal of maintaining heart rates >150 beats per minute (BPM). Real-time heart rate monitoring and rate of perceived exertion are used to monitor and document exercise intensity throughout the program. This exercise intensity was selected for the established effects on improving metabolic health in obese youth [49]. Resistance exercise includes circuit training using age and size appropriate equipment. Our previous studies suggest this form of exercise is both enjoyable and metabolically beneficial for obese youth [50]. In addition to structured PA classes, youth are ‘prescribed’ an additional day of unstructured PA of at least 60-minutes with a family member or peer in the program. This allows for flexibility in pursuing preferred activities that can be completed at the YMCA or elsewhere in the community to promote social support, role modeling, bonding among youth and families, and facilitate sustainability of a physically active lifestyle once the research study is completed. PA dosage will be assessed by measuring exercise intensity by heart rate and time spent above 150 BPM on a weekly basis. Intervention session themes and objectives are presented in Table 1.

**Table 1:** Sample of intervention session themes

Nutrition	Wellness	Physical Activity
Getting Started	Building Self-Esteem	Fuel Up with Cardio!
Health Awareness	Exploring Who I Am	Enjoy Being Fit
Champions in Eating	Anxiety	En Familia: Enjoying the Healthier Side of Fitness!
Roles and Responsibilities	Self-Nurturing	Exercise is Energy!
Family Dynamics	Identifying and Managing Feelings	Perseverance
How Sweet Are You?	Negative Self-Talk /Body-Talk	Teamwork
Cooking with Flavor	Staying Grounded	I am a Healthy Role-Model
Slim the Fat	Interpersonal Boundaries	My Exercise Role Model
Fast Food	Making Responsible Choices	Explore the World of Sports
Snack Attack		Motivate as Easy as 1, 2, 3
Culture and Health		Living as a Fit Unit!
Stay Strong		Let’s Go after Our Goals!
Building Family Communication		United and Stronger!
Shopping for Health		Redefine My Exercise Goals + Assessments

### Usual care control

Participants randomized to the UCC condition meet with a pediatric endocrinologist to review laboratory results and meet with a registered dietitian who will provide general lifestyle counseling on healthy eating and PA at baseline and 6-months. Randomizing obese youth with prediabetes to a true control condition is not ethical as rapid conversion from prediabetes to overt T2D can occur in a very short time frame. Therefore, the UCC arm designed for this study mirrors local standards/current practice for obese youth referred to the weight management program at our local children’s hospital. Upon completion of the study, control youth will be offered a 1-year membership to the YMCA. Although it would be preferable to offer the intervention to UCC youth, this is not feasible due to costs and time constraints.

### Booster sessions

Three booster sessions (months 7, 8, and 9) following the completion of the intensive lifestyle intervention will be held to support the maintenance of healthy lifestyle behaviors, address any challenges encountered, and promote successes achieved. Post-intervention clinical measures will be returned to participants at the first session and changes in health status will be discussed in the context of maintaining healthy lifestyle behaviors.

### Post-intervention follow-up

All youth, regardless of group, will be contacted on a monthly basis via phone, text, or email to enhance retention and ensure availability for testing 12-months after baseline.

### Primary outcomes (Table 2)

**Table 2:** Outcomes to be measured

Outcome	Instrument/Measurement
Glucose Tolerance	75gram, 2-hour Oral Glucose Tolerance Test
Insulin Sensitivity	Whole-Body Insulin Sensitivity Index
Insulin Secretion	Insulinogenic Index
β-cell function	Oral Disposition Index
Quality of Life	Youth Quality of Life, Weight-Specific Quality of Life
Total body composition (fat, muscle, and bone)	Dual-energy X-Ray Absorptiometry
Whole-abdominal fat distribution (subcutaneous abdominal adipose tissue, visceral adipose tissue, organ fat)	Magnetic Resonance Imaging
Physical Activity	3 Day Physical Activity Recall
Cardiorespiratory Fitness (Vo2peak)	Submaximal Exercise Test
Dietary Intake	2007 Block Food Screener

#### Glucose tolerance and insulin sensitivity (Aim 1)

Glucose tolerance and insulin sensitivity will be assessed by a 75-gram oral glucose tolerance test (OGTT) with multiple blood sampling for insulin glucose. An OGTT is preferred over fasting measures for differentiating T2D risk in youth and thus, offers a direct benefit to participants who may be at high risk for T2D [51]. Blood samples will be collected from an in-dwelling catheter at −15′, −5′, 30′, 60′, 90′ and 120′ post glucose consumption for measurement of plasma glucose and insulin. For the purposes of this study, glycemic status will be defined as “Normal” (fasting glucose <100 mg/dl and 2-hr glucose <120 mg/dl), “Prediabetic” (fasting glucose ≥100 mg/dl or 2-hr glucose ≥120 mg/dl), or “Diabetic” (fasting glucose ≥126 mg/dl or 2-hr glucose ≥200 mg/dl). Youth identified as “diabetic” at any point in the study will be referred for follow-up care and given the option to continue with the study once they have obtained clearance from their physician. Improvements in glucose tolerance will be assessed by decreases in 2-hour glucose levels while changes in insulin sensitivity will be estimated by the whole-body insulin sensitivity index (WBISI) [52]. The WBISI derived from an OGTT provides an integrated estimate of insulin sensitivity in youth that is highly correlated with the gold-standard hyperinsulinemic-euglycemic clamp (*r* = 0.78, *p* < 0.0005), is considered less risky than the clamp, and may be better tolerated in the pediatric population [53]. We have observed that the WBISI derived from an OGTT is more sensitive than fasting measures for assessing changes in insulin sensitivity among obese Latino adolescents participating in lifestyle interventions [54].

In addition to glucose tolerance and insulin sensitivity, insulin secretion will be estimated by the insulinogenic index defined as the ratio of the incremental change in insulin to glucose from fasting to 30′ (ΔInsulin 30′-fasting /ΔGlucose 30′-fasting) [55]. The insulinogenic index is considered a surrogate measure of early-phase insulin secretion and correlates reasonably well with first-phase insulin response as measured by the hyperglycemic clamp in youth (*r* = 0.56, *p* = 0.004) [56]. β-cell function will be estimated by the oral disposition index as the product of insulin sensitivity (WBISI) and insulin secretion (insulinogenic index) [57]. The disposition index is a measure of β-cell compensatory capacity that precedes and predicts the development of T2D and is thought to be an important physiologic measure for assessing changes in T2D risk over time [58, 59].

#### Quality of life (Aim 2)

Quality of Life (QoL) will be assessed using the Youth Quality of Life (YQOL) inventory. The YQOL was developed through semi-structured interviews with youth regarding positive and negative aspects of QoL [60]. Domains of self (e.g. feelings about one’s self), social relationships (e.g. friends and family), environment (e.g. social and cultural milieu) are assessed and an overall QoL score is computed. The instrument shows strong psychometric properties including internal consistency (Cronbach’s alpha >0.80), test-retest reliability (ICC >0.74), and construct validity with other pediatric QoL measures (*r* = 0.73, *P* < 0.05 with KINDL). Weight-specific QoL will be assessed by the YQOL-W which, measures three domains of weight-related QoL (Self, Social, and Environmental). It is specific to obese adolescents (11–18 years) for use in evaluating weight management interventions in clinical and community research [61, 62]. The instrument shows good reliability (ICC = 0.77) and construct validity with the children’s depression inventory (*r* = 0.57, *P* < 0.01) in adolescents and is more sensitive than generic measures for detecting changes in QoL among obese youth participating in lifestyle interventions [60].

#### Total body composition (Aim 3)

Total body composition (fat, muscle, and bone) will be assessed by Dual-energy X-Ray Absorptiometry (DXA) using the GE Lunar iDXA (GE Lunar, Madison, WI). DXA provides reasonable estimates of total body composition as well as changes in fat mass and lean tissue mass following lifestyle intervention in obese youth [63, 64]. A urine pregnancy test will be performed in females and a negative test is required before each DXA measurement. Pregnant females will be ineligible to participate in the study. Whole-abdominal fat distribution including subcutaneous abdominal adipose tissue volume, visceral adipose tissue volume, and organ fat (liver fat and pancreatic fat content) using advanced chemical-shift-encoded (CSE) water-fat magnetic resonance imaging will be used [65–68]. Post-processing of the subsequent CSE fat fraction data will be completed using a commercial semi-automated software tool (SliceOmatic; Tomovision, Inc., Montreal, Québec, Canada).

#### Physical activity/fitness assessment

PA will be measured using the 3 Day PA Recall, (3DPAR), an interviewer-administered recall instrument that measures the type of PA performed during the past 3 days (e.g. Tues, Mon, Sun). The 3DPAR allows for assessment of time spent in sedentary behaviors and types of activity that can be useful to identify differences in PA patterns between adolescents [69]. This instrument has shown good reliability (*r* = 0.67–0.83) and lower-to-moderate validity *r* = 0.29–0.64 when compared to accelerometer measured PA in youth [70, 71]. Cardiorespiratory fitness (Vo2peak) will be estimated by a submaximal exercise test developed and validated for obese youth [72]. Participants walk on a treadmill at a self-selected speed at 0% grade for 4 min. The grade is then increased to 5% while speed is maintained for 4 more minutes. Heart rate is recorded at the end of the 8 min and entered into the prediction equation.

#### Diet assessment

Dietary intake will be measured using the 2007 Food Block Screener for Ages 2–17. This 41-item screener assesses foods eaten during the previous week and was designed to identify dietary intake by food group [73]. National dietary surveys were used to inform the food selections to query, as well as to identify appropriate portion sizes and nutrient composition. This screener includes items commonly consumed by Latino youth and has been used to assess changes in dietary factors in Latino youth [74].

### Data analyses

#### Power analyses

We conducted power calculations for the ANCOVA’s that form the primary methods of analysis for Specific Aim 1 using preliminary data from an ongoing NIH-funded randomized control trial in a similar population. All power analyses used an alpha level of 0.05. For the ANCOVA’s (Specific Aim 1), we examined power to detect a large effect of a single predictor, after partialling out a single covariate (e.g., pretest outcome score). We assume that the effect size to be detected is large as defined by Cohen based on the preliminary intervention effect sizes for reductions in 2-hour glucose among prediabetic adolescents in our current study (Cohen’s d = 1.25). Power to detect a large effect of the intervention on improving glucose tolerance with an *N* = 100 is 0.85. These conservative power analyses indicate that we will have adequate power to detect large effect sizes using the ANCOVA models under Specific Aims 1 and 2 (effect size for change QoL in prediabetic youth in our current study is also large d = 1.07). Mediation analyses proposed in Specific Aim 3 are exploratory.

#### Preliminary analyses

Data will be cleaned to identify data entry errors, outliers, and non-normally distributed variables. If missing data is found to be unrelated to any study variable and a substantial amount of data are missing through attrition, we will handle missing data using the Full Information Maximum Likelihood methodology, which provides more unbiased parameter estimates and standard errors which is superior to the use of listwise deletion [75–77]. If distributions of key variables are non-normal, bias-corrected bootstrap methods that are robust to violations of normality will be used. For pretest data, scale item inter-correlations and a scale reliabilities analysis will be conducted to assess each scale’s internal consistency using the Cronbach α coefficient. We will also examine the descriptive statistics for all items and scales. These psychometric analyses will retain all participants who were assigned to the intervention or UCC groups (i.e., intent to treat).

#### Aim 1 analyses

We will test the hypothesis that adolescents who complete the intervention will exhibit significantly greater short-term and long-term improvements in glucose tolerance and increases in insulin sensitivity compared to UCC. ANCOVA models will use pretest measures as a set of covariates. Then we will conduct comparisons of effects for the intervention group relative to the control group (i.e., for the Intervention vs. UCC). Separate analyses will be conducted at the 6- and 12-month observation points. We will explore interactions between the covariate (baseline measure) and group (Intervention vs. UCC) to determine whether adolescents with lower baseline scores benefitted differentially from this program. Assuming that no interaction effects are found, we will examine the main effect of group assignment for each of the outcome measures, after adjusting for baseline scores. Analyses will be repeated for other T2D related outcomes (e.g. β-cell function). We will also conduct repeated measures ANCOVA models to examine differences in the effect of the intervention on primary outcomes at the 6- and 12-month time points.

#### Aim 2 analyses

We will use the same ANCOVA models described above to examine the effects of the intervention on general and weight-specific QoL. Results will indicate whether adolescents who were randomized to the intervention report greater increases in QoL compared to adolescents randomized to UCC.

#### Aim 3 analyses

We will also explore the mediating effects of changes in body mass, total body composition (fat mass and lean mass), regional body composition (visceral fat), and organ fat (liver and pancreatic fat) on changes in T2D risk markers by path analysis [75]. Each mediation model will include three variables: (1) experimental group membership represented by indicator variables, (2) a mediator measure (e.g., fat mass), and (3) an outcome measure (e.g., glucose tolerance). The model specifies direct paths from group membership to the mediator and outcome. Path coefficients and standard errors for these paths will be estimated. Effect size measures will include: (1) direct effects on the outcome and (2) mediated or indirect effects on the outcome. The latter will be estimated using path coefficient estimates with standard errors [78].

#### Aim 4 analyses

To examine the cost effectiveness of the intervention, we will estimate the incremental cost-effectiveness ratio (ICER) of the intervention compared to UCC based on changes in 2-hour glucose. The cost effectiveness analyses will be conducted from the societal perspective using 2-hour glucose at 12 months, direct medical costs, and non-medical costs. Direct medical costs include: personnel, intervention materials, MD and RD visits, lab tests, and procedures. Direct medical costs will be determined by grant related expenses and yearly financial reports. The non-medical costs will be participant time for travel estimated at $15 per hour, productivity loss, as determined by income and hours of work missed by parents, and commercial services for PA and nutrition instruction which will be invoiced. Base case and sensitivity analyses will be conducted using TreeAge software. The base case analysis model will use 6-month intervention and booster session costs and glucose tolerance at 12-months. ICERs will be calculated by dividing incremental costs by incremental effectiveness (change in 2- hour glucose). These results will provide further context about the cost associated with improving glucose tolerance and associated potential health benefits. Costs collected throughout the study will be inflated based on the published inflation rates in US dollars at the end of the study. Sensitivity analyses will be modeled based on those carried out for the DPP lifestyle intervention and we will assume adherence will decrease by 20% after 6-months [79].

## Discussion

Current annual costs of T2D in the U.S. are about $250 billion dollars [80]. Youth onset T2D is estimated to reduce life expectancy by 15 years creating burden for the workforce, economy, and health care system [81]. The Diabetes Prevention Program has been successful in reducing diabetes risk factors in high-risk adults, yet it has not been adapted and tested with high-risk youth from any population [82]. Accordingly, the present study addresses this important gap in diabetes prevention, while also bridging the gap between science and practice in testing the short- and long-term efficacy of a culturally-adapted diabetes prevention program curriculum with a sample of obese Latino youth with prediabetes.

This study addresses disparities in T2D using an innovative approach that delivers a community-based, culturally-grounded intervention to a vulnerable and key population subgroup. This lifestyle intervention is highly innovative in that it is adapted for responsiveness to age and ethno-cultural background. It also includes an emotional health component and is delivered in a community setting using a family-based model. This intervention’s enhancement of emotional and psychosocial health may offer the most relevant and readily perceived benefit for the obese youth who participate in this lifestyle enhancement intervention. Based on this comprehensive curriculum, this prevention intervention could produce sustained long-term changes in health behaviors, thus contributing significantly to T2D risk reduction [83]. This can be attained by engaging these families in prevention and treatment, which is considered the “gold-standard” for facilitating behavior change in youth [32, 84].

Interventions for obese youth are often implemented in a clinical or school-based setting. However, these settings may limit the size, scope, and generalizability of the study, while also presenting challenges for fully integrating family participation [85]. In contrast, interventions implemented within high-risk communities offer an innovative approach to diabetes prevention that can also benefit the populations that need it the most [86]. Implementing this intervention in collaboration with the YMCA, leverages this community as a resource not only as a delivery site, but also as a location for providing local community residents with an opportunity to engage in healthy behaviors on a daily basis. Partnering with the YMCA also provides opportunities for the institutionalization of a diabetes prevention program that is tailored to high-risk, minority youth. Furthermore, the YMCA’s Healthier Communities Initiative supports efforts that focus on making policy and environmental changes that support healthy lifestyles in communities across the United States. This academic-community partnership serves as a natural extension of the Healthier Communities initiative that can further enhance the sustainability and scalability of this diabetes prevention intervention [87].

### Implications of findings

This study aims to address critical gaps in how T2D prevention interventions for high-risk youth are implemented and evaluated, thus offering the potential for the direct translation of this intervention, and the promise of promoting broad, solution-oriented policies and programs. Findings from this study can be used to establish a model lifestyle intervention for diabetes prevention among Latino youth and their families, as this intervention can be translated on a wide-scale and applied to other at-risk minority populations. Furthermore, this study will calculate the incremental cost effectiveness of this intervention for improving glucose tolerance and increasing insulin sensitivity, two important indicators that prelude T2D. We will be able to project these data forward based on 2-hour glucose changes as to the probability of developing diabetes and the long term impact on health related quality of life. This type of additional analysis would allow us to express the results in terms of dollars spent per quality-adjusted life year gained and this is easier to interpret for policy and guidelines than simply using the dollar spent per change in clinical measure. National statistics and future projections for the incidence of T2D among Latino youth highlight the clear economic need for cost-effective T2D prevention programs developed for obese Latino youth who are affected by prediabetes. This study will also provide economic evidence that can be used in partnership with policymakers to leverage policy support for diabetes prevention programming.

### Potential limitations

This study is limited by its sample that consists of obese Latino adolescents, given its focus on these youths based on compelling evidence that this population is at increased risk for T2D. Although the present study design limits generalizability, we anticipate that this basic framework can be adapted for implementation with at-risk youth from other racial/ethnic groups. Focusing exclusively on Latino youth will allow us to apply the most relevant delivery strategy to a culturally similar population of youth for a rigorous test of intervention efficacy. Furthermore, using an OGTT to simultaneously assess glucose tolerance and other T2D markers (insulin sensitivity and β-cell function) constitutes a strength as well as a limitation. Although the OGTT is the preferred measure for comprehensive assessment of T2D in youth and provides physiologically relevant estimates of T2D risk, it is not a gold-standard assessment for insulin action or secretion [51, 88–90]. We have considered other measures of T2D risk such as fasting surrogates (e.g., HOMA-IR), and the hyperinsulinemic-euglycemic clamp. However, fasting surrogates may not be sufficiently sensitive to measure changes following lifestyle interventions in youth [54]. Furthermore, the clamp is costly, invasive, and difficult to perform in an outpatient setting [54, 91, 92].

## Conclusion

There is critical need for T2D prevention research focused on high-risk youth including those from minority and low socioeconomic groups where diabetes is most prevalent [17, 93]. This study will collaborate with local community partnerships to adapt and further refine a diabetes prevention program for obese, prediabetic Latino youth. This study will deliver a culturally-grounded intervention in the YMCA that brings Latino families together to address a significant and pervasive public health problem during a critical developmental period. This study may yield efficacious results that would allow this intervention to be disseminated to other Latino communities, to expand its public health impact for prevention or delaying the onset of T2D, while doing so in a cost-effective manner.

## Abbreviations

BPM: Beats per minute
CSE: Chemical-shift-encoded
DPP: Diabetes prevention program
EEM: Expanded Ecodevelopmental model
ICER: Incremental cost-effectiveness ratio
OGTT: Oral glucose tolerance test
PA: Physical activity
QoL: Quality of life
RCT: Randomized controlled trial
SCT: Social Cognitive Theory
T2D: Type 2 diabetes
UCC: Usual care control
WBISI: Whole-body insulin sensitivity index

## References

[1] Huang TT, Goran MI (2003). Prevention of type 2 diabetes in young people: a theoretical perspective. Pediatr Diabetes.

[2] Ogden CL (2010). Prevalence of high body mass index in US children and adolescents, 2007-2008. JAMA.

[3] Pettitt DJ (2014). Prevalence of diabetes in U.S. youth in 2009: the SEARCH for diabetes in youth study. Diabetes Care.

[4] Menke A, Casagrande S, Cowie CC (2016). Prevalence of diabetes in adolescents aged 12 to 19 years in the United States, 2005-2014. JAMA.

[5] Lawrence JM (2009). Diabetes in Hispanic American youth: prevalence, incidence, demographics, and clinical characteristics: the SEARCH for diabetes in youth study. Diabetes Care.

[6] Narayan KM (2003). Lifetime risk for diabetes mellitus in the United States. JAMA.

[7] Goran MI (2002). Insulin resistance and associated compensatory responses in african-american and Hispanic children. Diabetes Care.

[8] Lee JM (2006). Prevalence and determinants of insulin resistance among U.S. adolescents: a population-based study. Diabetes Care.

[9] Goran MI, Ball GD, Cruz ML (2003). Obesity and risk of type 2 diabetes and cardiovascular disease in children and adolescents. J Clin Endocrinol Metab.

[10] Goran MI, Gower BA (2001). Longitudinal study on pubertal insulin resistance. Diabetes.

[11] Weiss R (2005). Predictors of changes in glucose tolerance status in obese youth. Diabetes Care.

[12] Duncan GE (2006). Prevalence of diabetes and impaired fasting glucose levels among US adolescents: National Health and Nutrition Examination Survey, 1999-2002. Arch Pediatr Adolesc Med.

[13] Zeller MH, Modi AC (2006). Predictors of health-related quality of life in obese youth. Obesity (Silver Spring).

[14] Ul-Haq Z (2013). Meta-analysis of the association between body mass index and health-related quality of life among children and adolescents, assessed using the pediatric quality of life inventory index. J Pediatr.

[15] Franks PW (2010). Childhood obesity, other cardiovascular risk factors, and premature death. N Engl J Med.

[16] Census US (2012). Most children younger than age 1 are minorities, Census Bureau Reports.

[17] Haemer MA (2014). Addressing prediabetes in childhood obesity treatment programs: support from research and current practice. Child Obes.

[18] Lee DC, Sui X, Blair SN (2009). Does physical activity ameliorate the health hazards of obesity?. Br J Sports Med.

[19] Knowler WC (2002). Reduction in the incidence of type 2 diabetes with lifestyle intervention or metformin. N Engl J Med.

[20] Kitabchi AE (2005). Role of insulin secretion and sensitivity in the evolution of type 2 diabetes in the diabetes prevention program: effects of lifestyle intervention and metformin. Diabetes.

[21] Group TS (2013). Retinopathy in youth with type 2 diabetes participating in the TODAY clinical trial. Diabetes Care.

[22] Group TS (2013). Rapid rise in hypertension and nephropathy in youth with type 2 diabetes: the TODAY clinical trial. Diabetes Care.

[23] Group TS (2013). Lipid and inflammatory cardiovascular risk worsens over 3 years in youth with type 2 diabetes: the TODAY clinical trial. Diabetes Care.

[24] Whitlock EP (2010). Effectiveness of weight management interventions in children: a targeted systematic review for the USPSTF. Pediatrics.

[25] Seo DC, Sa J (2010). A meta-analysis of obesity interventions among U.S. minority children. J Adolesc Health.

[26] Ho M (2012). Effectiveness of lifestyle interventions in child obesity: systematic review with meta-analysis. Pediatrics.

[27] Epstein LH (1998). Treatment of pediatric obesity. Pediatrics.

[28] Flynn MA (2006). Reducing obesity and related chronic disease risk in children and youth: a synthesis of evidence with ‘best practice’ recommendations. Obes Rev.

[29] Flores G (1998). Access barriers to health care for Latino children. Arch Pediatr Adolesc Med.

[30] Franks PW, Pearson E, Florez JC (2013). Gene-environment and gene-treatment interactions in type 2 diabetes: progress, pitfalls, and prospects. Diabetes Care.

[31] Flores G (2002). The health of Latino children: urgent priorities, unanswered questions, and a research agenda. JAMA.

[32] Davison KK, Lawson HA, Coatsworth JD (2012). The Family-centered Action Model of Intervention Layout and Implementation (FAMILI): the example of childhood obesity. Health Promot Pract.

[33] Castro FG, Shaibi GQ, Boehm-Smith E (2009). Ecodevelopmental contexts for preventing type 2 diabetes in Latino and other racial/ethnic minority populations. J Behav Med.

[34] Hill JO (2013). Scientific statement: Socioecological determinants of prediabetes and type 2 diabetes. Diabetes Care.

[35] Baker MK (2011). Behavioral strategies in diabetes prevention programs: a systematic review of randomized controlled trials. Diabetes Res Clin Pract.

[36] Diabetes Prevention Program Research Group (2002). The Diabetes Prevention Program (DPP): description of lifestyle intervention. Diabetes Care.

[37] Task Force on Community Preventive, S (2002). Recommendations to increase physical activity in communities. Am J Prev Med.

[38] Wang Y, Wu Y, Wilson RF, et al. Childhood Obesity Prevention Programs: Comparative Effectiveness Review and Meta-Analysis [Internet]. Rockville (MD): Agency for Healthcare Research and Quality (US). 2013. (Comparative Effectiveness Reviews, No. 115.) Available from: https://www.ncbi.nlm.nih.gov/books/NBK148737/. 23865092

[39] Shaibi GQ (2015). Diabetes Prevention for Latino Youth: Unraveling the Intervention “Black Box”. Health Promot Pract.

[40] Giannini C (2012). Evidence for early defects in insulin sensitivity and secretion before the onset of glucose dysregulation in obese youths: a longitudinal study. Diabetes.

[41] American Diabetes Association. Diagnosing diabetes and learning about prediabetes. 2016. Available from: http://www.diabetes.org/diabetes-basics/diagnosis/?referrer. Accessed Nov 2016.

[42] Shaibi GQ (2012). Effects of a culturally grounded community-based diabetes prevention program for obese Latino adolescents. Diabetes Educ.

[43] Shaibi GQ (2010). Development, implementation, and effects of community-based diabetes prevention program for obese latino youth. J Prim Care Community Health.

[44] Williams AN (2016). Culturally-grounded diabetes prevention program for obese Latino youth: Rationale, design, and methods.

[45] Castro FG, Alarcon EH (2002). Integrating cultural factors into drug abuse prevention and treatment with racial/ethnic minorities. J Drug Issues.

[46] Keller C, Coe K, Shiabi GS (2015). Using rituals for intervention refinement. Health, Culture, and Society.

[47] Frieden TR, C. Centers for Disease, and Prevention (2014). Strategies for reducing health disparities - selected CDC-sponsored interventions, United States, 2014. Foreword. MMWR Suppl, 2014.

[48] Institute of Medicine. 2008. Challenges and Successes in Reducing Health Disparities: Workshop Summary. Washington, DC: The National Academies Press. https://doi.org/10.17226/12154. 25009895

[49] Gutin B (2002). Effects of exercise intensity on cardiovascular fitness, total body composition, and visceral adiposity of obese adolescents. Am J Clin Nutr.

[50] Benson AC, Torode ME, Fiatarone Singh MA (2008). Effects of resistance training on metabolic fitness in children and adolescents: a systematic review. Obes Rev.

[51] Kaufman FR (2005). Screening for abnormalities of carbohydrate metabolism in teens. J Pediatr.

[52] Matsuda M, DeFronzo RA (1999). Insulin sensitivity indices obtained from oral glucose tolerance testing: comparison with the euglycemic insulin clamp. Diabetes Care.

[53] Yeckel CW (2004). Validation of insulin sensitivity indices from oral glucose tolerance test parameters in obese children and adolescents. J Clin Endocrinol Metab.

[54] Shaibi GQ (2011). Improving insulin resistance in obese youth: choose your measures wisely. Int J Pediatr Obes.

[55] Phillips DI (1994). Understanding oral glucose tolerance: comparison of glucose or insulin measurements during the oral glucose tolerance test with specific measurements of insulin resistance and insulin secretion. Diabet Med.

[56] Bacha F, Gungor N, Arslanian SA (2008). Measures of beta-cell function during the oral glucose tolerance test, liquid mixed-meal test, and hyperglycemic clamp test. J Pediatr.

[57] Bergman RN, Phillips LS, Cobelli C (1981). Physiologic evaluation of factors controlling glucose tolerance in man: measurement of insulin sensitivity and beta-cell glucose sensitivity from the response to intravenous glucose. J Clin Invest.

[58] Bergman RN (2002). Accurate assessment of beta-cell function: the hyperbolic correction. Diabetes.

[59] Utzschneider KM (2009). Oral disposition index predicts the development of future diabetes above and beyond fasting and 2-h glucose levels. Diabetes Care.

[60] Patrick DL, Edwards TC, Topolski TD (2002). Adolescent quality of life, part II: initial validation of a new instrument. J Adolesc.

[61] Morales LS (2011). Measurement properties of a multicultural weight-specific quality-of-life instrument for children and adolescents. Qual Life Res.

[62] Patrick DL (2011). Weight loss and changes in generic and weight-specific quality of life in obese adolescents. Qual Life Res.

[63] Helba M, Binkovitz LA (2009). Pediatric body composition analysis with dual-energy X-ray absorptiometry. Pediatr Radiol.

[64] Wells JC (2010). Evaluation of DXA against the four-component model of body composition in obese children and adolescents aged 5-21 years. Int J Obes (Lond).

[65] Hu HH, Bornert P, Hernando D (2012). ISMRM workshop on fat-water separation: insights, applications and progress in MRI. Magn Reson Med.

[66] Hu HH (2010). Comparison of fat-water MRI and single-voxel MRS in the assessment of hepatic and pancreatic fat fractions in humans. Obesity (Silver Spring).

[67] Hu HH (2014). MRI detection of brown adipose tissue with low fat content in newborns with hypothermia. Magn Reson Imaging.

[68] Hu HH (2013). Comparison of brown and white adipose tissues in infants and children with chemical-shift-encoded water-fat MRI. J Magn Reson Imaging.

[69] Pratt C (2008). Sedentary activity and body composition of middle school girls: the trial of activity for adolescent girls. Res Q Exerc Sport.

[70] McMurray RG (2004). Comparison of two approaches to structured physical activity surveys for adolescents. Med Sci Sports Exerc.

[71] Pate RR (2003). Validation of a 3-day physical activity recall instrument in female youth. Pediatr Exerc Sci.

[72] Nemeth BA (2009). Submaximal treadmill test predicts VO2max in overweight children. J Pediatr.

[73] Hunsberger M (2015). Relative validation of Block Kids Food Screener for dietary assessment in children and adolescents. Matern Child Nutr.

[74] Davis JN (2011). LA Sprouts: a gardening, nutrition, and cooking intervention for Latino youth improves diet and reduces obesity. J Am Diet Assoc.

[75] Enders, CK. The relative performance of full-information maximum likelihood estimation for missing data in structural equation models. DigitalCommons@University of Nebraska-Lincoln; 1999

[76] Schafer JL (1997). Analysis of incomplete data.

[77] Schafer JL, Graham JW (2002). Missing data: Our view of the state of the art. Psychol Methods.

[78] Baron RM, Kenny DA (1986). The moderator-mediator variable distinction in social psychological research: conceptual, strategic, and statistical considerations. J Pers Soc Psychol.

[79] Herman WH (2005). The cost-effectiveness of lifestyle modification or metformin in preventing type 2 diabetes in adults with impaired glucose tolerance. Ann Intern Med.

[80] American Diabetes Association (2012). Economic costs of diabetes in the U.S. in 2012. Diabetes Care.

[81] Rhodes ET (2012). Health-related quality of life in adolescents with or at risk for type 2 diabetes mellitus. J Pediatr.

[82] Jackson L (2009). Translating the diabetes prevention program into practice: a review of community interventions. Diabetes Educ.

[83] Ferguson MA (1999). Effects of exercise training and its cessation on components of the insulin resistance syndrome in obese children. Int J Obes Relat Metab Disord.

[84] Campbell KJ, Hesketh KD (2007). Strategies which aim to positively impact on weight, physical activity, diet and sedentary behaviours in children from zero to five years. A systematic review of the literature. Obes Rev.

[85] Sobol-Goldberg S, Rabinowitz J, Gross R (2013). School-based obesity prevention programs: a meta-analysis of randomized controlled trials. Obesity (Silver Spring).

[86] Whittemore R (2011). A systematic review of the translational research on the Diabetes Prevention Program. Transl Behav Med.

[87] Adamson K (2009). The YMCA/Steps Community Collaboratives, 2004-2008. Prev Chronic Dis.

[88] Weyer C (2001). Insulin resistance and insulin secretory dysfunction are independent predictors of worsening of glucose tolerance during each stage of type 2 diabetes development. Diabetes Care.

[89] Retnakaran R (2008). Hyperbolic relationship between insulin secretion and sensitivity on oral glucose tolerance test. Obesity (Silver Spring).

[90] Weiss R, Caprio S (2006). Development of type 2 diabetes in children and adolescents. Minerva Med.

[91] Kester LM, Hey H, Hannon TS (2012). Using hemoglobin A1c for prediabetes and diabetes diagnosis in adolescents: can adult recommendations be upheld for pediatric use?. J Adolesc Health.

[92] Haymond MW (2003). Measuring insulin resistance: a task worth doing. But how?. Pediatr Diabetes.

[93] Linder BL, Fradkin JE, Rodgers GP (2013). The TODAY study: an NIH perspective on its implications for research. Diabetes Care.

